# Epidemiological and laboratory characteristics of Omicron infection in a general hospital in Guangzhou: a retrospective study

**DOI:** 10.3389/fpubh.2023.1289668

**Published:** 2023-11-29

**Authors:** Jingrou Chen, Yang Wang, Hongwei Yu, Ruizhi Wang, Xuegao Yu, Hao Huang, Lu Ai, Tianruo Zhang, Bin Huang, Min Liu, Tao Ding, Yifeng Luo, Peisong Chen

**Affiliations:** ^1^Department of Laboratory Medicine, The First Affiliated Hospital, Sun Yat-sen University, Guangzhou, China; ^2^Department of Radiation Hygiene and Protection, Guangdong Province Prevention and Treatment Center for Occupational Diseases, Guangzhou, China; ^3^Department of Medical Laboratory Technology, Medical College of Jiaying University, Meizhou, China; ^4^Department of Immunology and Microbiology, Zhongshan School of Medicine, Sun Yat-sen University, Guangzhou, China; ^5^Key Laboratory of Tropical Diseases Control, Ministry of Education, Sun Yat-sen University, Guangzhou, China; ^6^Division of Pulmonary and Critical Care Medicine, Institute of Respiratory Diseases, The First Affiliated Hospital, Sun Yat-sen University, Guangzhou, China

**Keywords:** Omicron, Ct value, sex, age, laboratory biomarkers

## Abstract

The COVID-19 pandemic caused by SARS-CoV-2 has emerged as a major global public health concern. In November 2022, Guangzhou experienced a significant outbreak of Omicron. This study presents detailed epidemiological and laboratory data on Omicron infection in a general hospital in Guangzhou between December 1, 2022, and January 31, 2023. Out of the 55,296 individuals tested, 12,346 were found to be positive for Omicron. The highest prevalence of positive cases was observed in the 20 to 39 age group (24.6%), while the lowest was in children aged 0 to 9 years (1.42%). Females had a higher incidence of infection than males, accounting for 56.6% of cases. The peak time of Omicron infection varied across different populations. The viral load was higher in older adults and children infected with Omicron, indicating age-related differences. Spearman’s rank correlation analysis revealed positive correlations between Ct values and laboratory parameters in hospitalized patients with Omicron infection. These parameters included CRP (*r_s_* = 0.059, *p* = 0.009), PT (*r_s_* = 0.057, *p* = 0.009), INR (*r_s_* = 0.055, *p* = 0.013), AST (*r_s_* = 0.067, *p* = 0.002), LDH (*r_s_* = 0.078, *p* = 0.001), and BNP (*r_s_* = 0.063, *p* = 0.014). However, EO (Eosinophil, *r_s_* = −0.118, *p* < 0.001), BASO (basophil, *r_s_* = −0.093, *p* < 0.001), and LY (lymphocyte, *r_s_* = −0.069, *p* = 0.001) counts showed negative correlations with Ct values. Although statistically significant, the correlation coefficients between Ct values and these laboratory indices were very low. These findings provide valuable insights into the epidemiology of Omicron infection, including variations in Ct values across gender and age groups. However, caution should be exercised when utilizing Ct values in clinical settings for evaluating Omicron infection.

## Introduction

The emergence of severe acute respiratory syndrome coronavirus 2 (SARS-CoV-2) has triggered a global health crisis (1). The virus quickly spread to over 200 countries, leading to widespread illness and fatalities (2), and underwent multiple mutations during the epidemic (3). Currently, the Omicron variant has become the predominant strain of SARS-CoV-2 worldwide (4, 5). Its heightened transmissibility and potential to evade immunity from prior infections or vaccinations have garnered significant attention (6–8). In contrast to previous variants such as Delta (9), Omicron has exhibited a higher incidence of breakthrough infections among vaccinated individuals (10). This led to an outbreak in Guangzhou from December 2022 to January 2023, prompting the Guangzhou Center for Disease Control and Prevention to disseminate information and guidance on managing Omicron. Despite vaccination campaigns, there remains a need for a deeper understanding of Omicron’s epidemiology. Although there have been reports in China (11), Omicron has been found to have a higher viral load, faster replication speed, and shorter duration. However, detailed statistics on the infection rates and peak times among different populations in the South China region have not yet been compiled. Using a comprehensive large hospital in Southern China as an observational window, we describe the rapid spread of Omicron in the region, highlighting its high transmissibility. This complements existing research findings on Omicron’s infection rates in China. The study aims to contribute to our understanding of Omicron’s epidemiological characteristics by investigating the relationship between infection rates and epidemiological features in different groups. Additionally, despite numerous studies and reports on the Ct values of the novel coronavirus, the correlation between Ct values and laboratory marker detection results has not been extensively described. Our research aims to establish, for the first time, the correlation between viral load (indicated by N gene Ct values) and other relevant laboratory markers. By examining these laboratory parameters, the study aims to provide insights into the association between viral load and clinical outcomes, shedding light on potential indicators of disease severity. Additionally, this large-scale outbreak of Omicron in Guangzhou city necessitates a comprehensive understanding of its epidemiological characteristics and its association with laboratory results.

Numerous studies have highlighted age and gender as influential factors in COVID-19 outcomes (12–14). The highest incidence of infection occurs among individuals aged 16–49, with a relatively higher incidence among females (15). Moreover, research has revealed that Omicron is more prevalent in Africa and Asia, and less common in Europe and North America (16). Recently, the use of cycle threshold (Ct) values of genes associated with Omicron infection has gained attention as a potential predictor of viral load (17–19). Additionally, previous reports have indicated that Ct values for COVID-19 correlate with serum biomarkers. Routine laboratory blood tests, including Eosinophils (EO) (20, 21), C-reactive protein (CRP) (22–24), interleukin 6 (IL-6) (25, 26), Prothrombin time (PT) (27), and lactate dehydrogenase (LDH) (21, 28), have been implicated in the pathogenesis of COVID-19 and are used as indicators of disease severity. However, there have been no reports exploring the correlation between recent Omicron variant strains and laboratory-related indicators. Therefore, this study aims to investigate the relationship between Ct values of relevant genes, age, sex, and laboratory biomarkers, with a focus on identifying the most relevant biomarkers that reflect changes in hematology, liver function, cardiac function, and coagulation after Omicron infection.

The findings of this study will contribute to the development of public health policies to manage this ongoing global health emergency. Additionally, they will provide valuable insights into the clinical diagnosis and evaluation of Omicron infection.

## Methods and materials

### Study design

This retrospective study aims to analyze the temporal patterns of the outbreak in Guangzhou and identify trends or changes over time. The study participants include hospitalized patients and individuals who voluntarily visited the hospital for testing within 3 days of experiencing suspected symptoms. The categorization of patients into these groups is aimed at better understanding the infection situations among different groups during the Omicron outbreak. Based on occupation, individuals can be divided into Medical Staff and Non-medical Staff. Non-medical Staff can be classified into three groups based on the progression of the disease: Societal, Outpatients, and Inpatients. Societal individuals are typically asymptomatic, Outpatients exhibit disease-related symptoms, and Inpatients experience severe conditions. We further divided the patients into four groups: (i) Societal, comprising individuals who voluntarily underwent nucleic acid testing (NAT) despite unclear symptoms or clinical manifestations; (ii) Medical Staff, consisting of hospital staff who voluntarily underwent NAT despite unclear clinical symptoms; (iii) Outpatients, comprising individuals who visited our hospital with respiratory symptoms or other suspicious symptoms or voluntarily requested NAT for other reasons; and (iv) Inpatients, comprising patients who were admitted due to Omicron infection or developed Omicron infection during their hospital stay. Additionally, we aim to investigate the correlation between Ct values of associated genes, age, sex, and routine laboratory blood tests.

### Data collection

The data for this study were collected from individuals who underwent NAT at our hospital from December 1, 2022, to January 31, 2023. Among them, patients infected with Omicron were selected based on positive results by real-time PCR. Demographic and clinical information, including age, sex, department, and laboratory results, were extracted from the medical records. Inclusion criteria for case selection are as follows: 1. Individuals who voluntarily participated in NAT or individuals who underwent NAT due to similar symptoms. 2. Patients with complete and reliable data information. Exclusion criteria include incomplete laboratory data, incomplete patient information, and ambiguous laboratory diagnoses.

### Ct value

Pharyngeal swab samples were collected following the Laboratory Testing Technical Guidelines for Novel Coronavirus Pneumonia. Trained nurses performed the sample collection using standard procedures. The samples were then subjected to RNA detection using the 2019-nCoV nucleic acid detection kit (DaAn Gene, China). The kit employs a one-step RT-PCR technique and specifically amplifies the ORF1ab and N genes of the novel coronavirus 2019-nCoV. Specific primers and fluorescent probes were designed to accurately detect the presence of novel coronavirus RNA in the specimens. The kit demonstrates outstanding precision and repeatability, consistent batch-to-batch performance, and high accuracy. With a minimum detection limit of 200 virus copies per milliliter, they exhibit exceptional sensitivity, providing crucial support for reliable detection of COVID-19 virus infections. Moreover, Da An’s Kit can complete the testing process rapidly, significantly reducing the turnaround time (29, 30). The kit has obtained certification and approval from the NMPA and is the most widely used test kit in China. RNA extraction was carried out using the Stream SP96 (DaAn Gene, China) fully automated nucleic acid extractor.

For the PCR reaction, a 20 μL amplification system was prepared, consisting of 17 μL of reaction solution A, 3 μL of reaction solution B, and 10 μL of the test nucleic acid. The amplification process was conducted on the ABI-7500 instrument (Thermo Fisher, USA) under specific reaction conditions: reverse transcription at 50°C for 2 min, pre-denaturation at 95°C for 2 min, denaturation at 95°C for 5 s, and annealing and extension at 60°C for 35 s (42 cycles). The resulting Ct value for each target gene was recorded, with a Ct value of <40 considered positive. Based on their Ct values, patients were categorized into three groups: ≤20 for high viral load, >20 and ≤30 for intermediate viral load, and >30 for low viral load (20).

### Routine laboratory biomarkers

During the SARS-CoV-2 testing process, laboratory biomarker samples were simultaneously collected. The blood routine indicators, including complete blood count (CBC) and platelets (PLT), were measured using the Mindray BC7500 instrument. The detection method used was Laser flow cytometry combined with fluorescence staining technology. The CRP was measured using the Latex-enhanced immune scattering turbidimetry method and detected using the Mindray 6,800 system.

Coagulation items, such as APTT, PT, INR, TT, FIB, and D-dimer, were measured using the Stago STAR Max instrument with the coagulation method.

Liver enzyme indicators (AST, ALT, LDH) were measured using the Rate method and detected using the Beckman Coulter AU5800.

Immunization program indicators (IL-6, CKMB, TNT, MYO, BNP, PCT) were measured using the Roche Cobas e801 equipment.

### Statistical analysis

Descriptive statistics are used to analyze the data, including calculating means, standard deviations, medians, and proportions. Time series analysis was performed to examine temporal patterns of the outbreaks and identify trends or changes over time.

We employed a chi-square test to assess potential variations in Ct values between genders. Additionally, we conducted a t-test to compare Ct values across different age groups, allowing us to evaluate any potential differences. Moreover, we utilized analysis of variance (ANOVA) to investigate potential disparities in various laboratory indicators among distinct Ct value groups.

To investigate the causal relationship between the intensity of various biological indicators and Ct values, serum biological indicators were divided into three groups based on N gene Ct values (>30, >20 ≤ 30, and ≤ 20). Due to the non-normal distribution of Ct values, we conducted the Wilcoxon rank-sum test to compare the medians of various biological indicators among the three groups. Additionally, since we categorized Ct values into three levels, the data falls into ordinal data. Therefore, to identify potential factors associated with viral load, we utilized Spearman rank correlation analysis to determine the correlation between Ct values and various serum biomarkers. Spearman’s rank correlation is an appropriate nonparametric estimator for estimating the correlation between two discrete variables. Spearman’s coefficient absolute value of *r_s_* in the range of 0.8–1 indicates a very strong correlation, 0.6–0.8 a strong correlation, 0.4–0.6 a medium correlation, and <0.4 a weak correlation (31). All statistical analyses were conducted using SPSS 26 and GraphPad Prism 8.0.

## Results

### Epidemiology of Omicron infection: age and temporal trends in disease incidence

**Figure 1:**
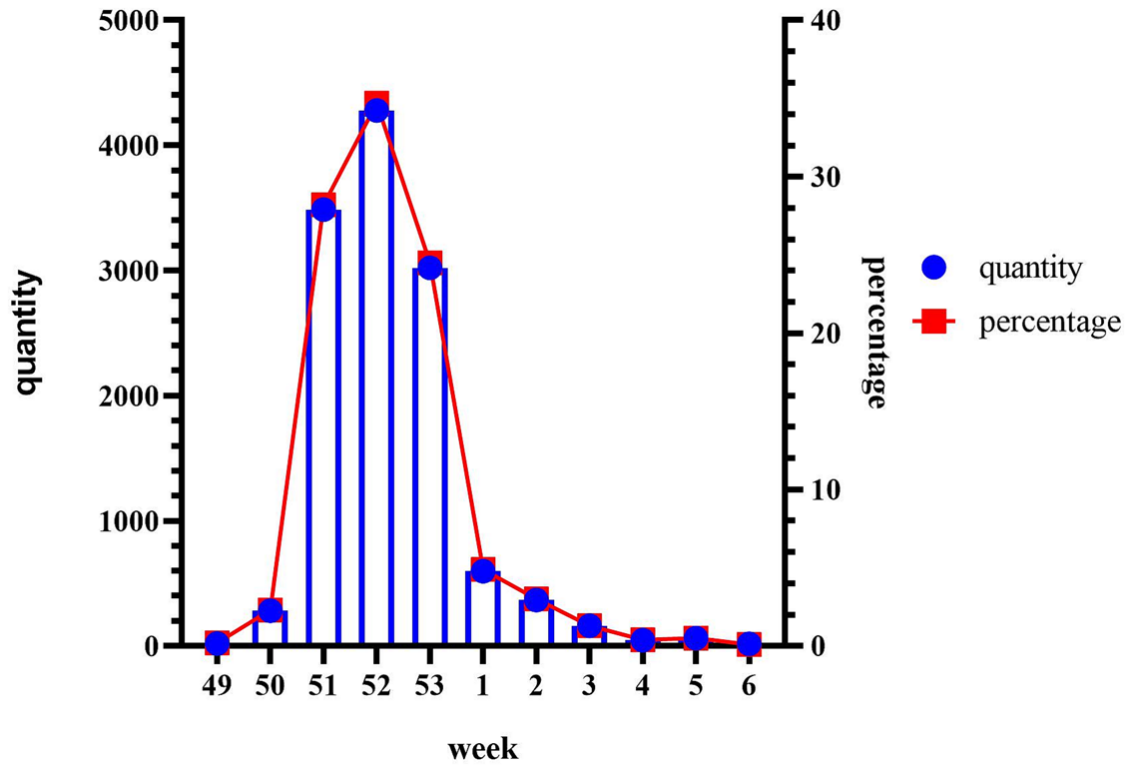
Cumulative weekly infection positivity and total during the outbreak. Collect data from December 1, 2022 (week 49) to January 31, 2023 (week 6), including the percentage of positive cases of SARS-CoV-2 in our hospital per week divided by the total number of people tested. The *y*-axis on the right represents the cumulative percentage of positive cases, while the *x*-axis corresponds to the weeks of the outbreak. The line graph illustrates the trend of infection positivity rate over time, and the bar chart represents the cumulative total.

**Figure 2:**
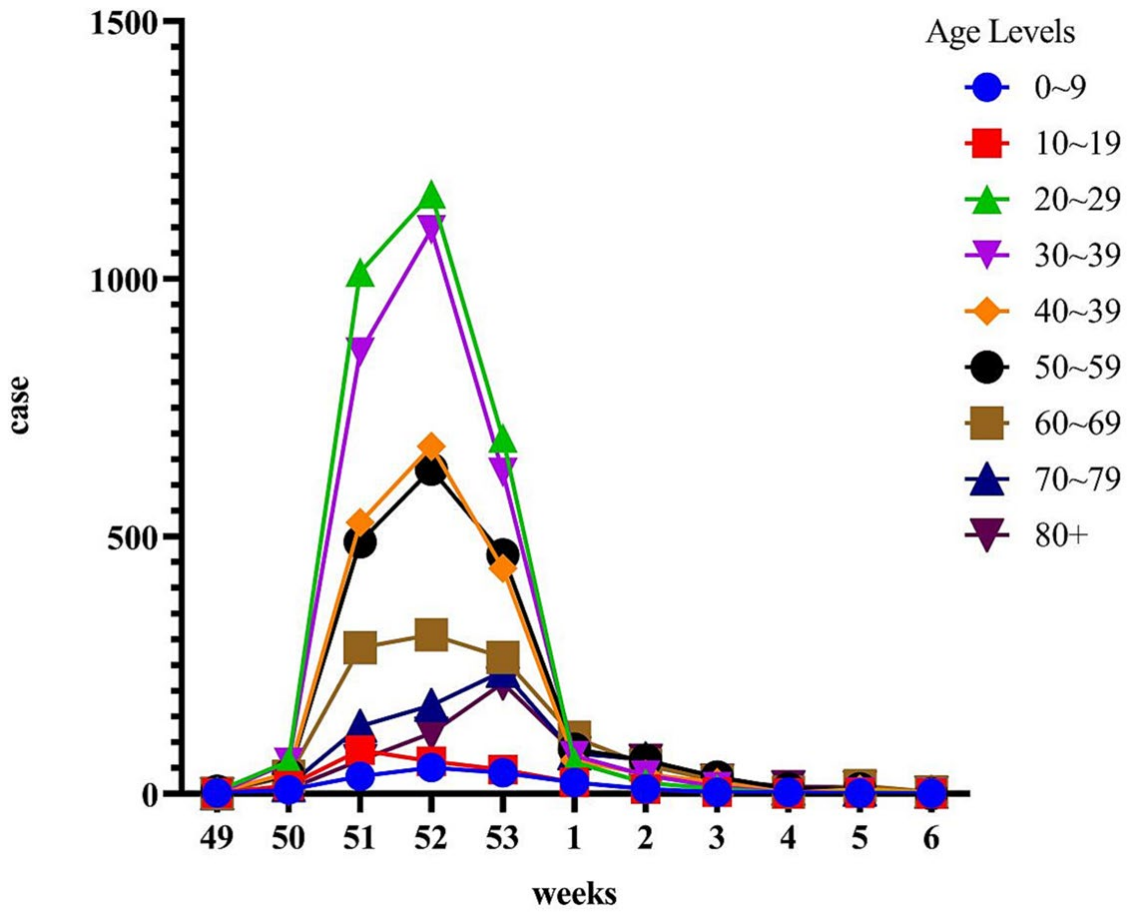
Time course of cumulative incidence by age group during the opening of the epidemic. The *x*-axis represents the period from week 49 to week 6, while the *y*-axis represents the cumulative incidence percentage. The age groups are categorized and indicated on the legend.

During the period from December 1, 2022, to January 31, 2023, data was collected from 55,296 patients who underwent Omicron infection testing. The average age of the included patients was 41.08 ± 17.87 years. The peak of positive cases occurred in the 52nd week, followed by a sharp decline in the 53rd week (Figure 1). We analyzed the cumulative incidence rates of specific age groups reported weekly. The results revealed a significant increase in case numbers across all age groups starting from the 50th week. By the 52nd week, the 20–39 age group had the highest number of cases, while the 0–9 age group had the fewest. However, the 70 and above age group reached its peak in the 53rd week. Following the peak, there was a continuous decline in all age groups in the first week of 2023 (Figure 2).

### Temporal analysis of Omicron outbreaks and peak incidence rates in different population categories

**Figure 3:**
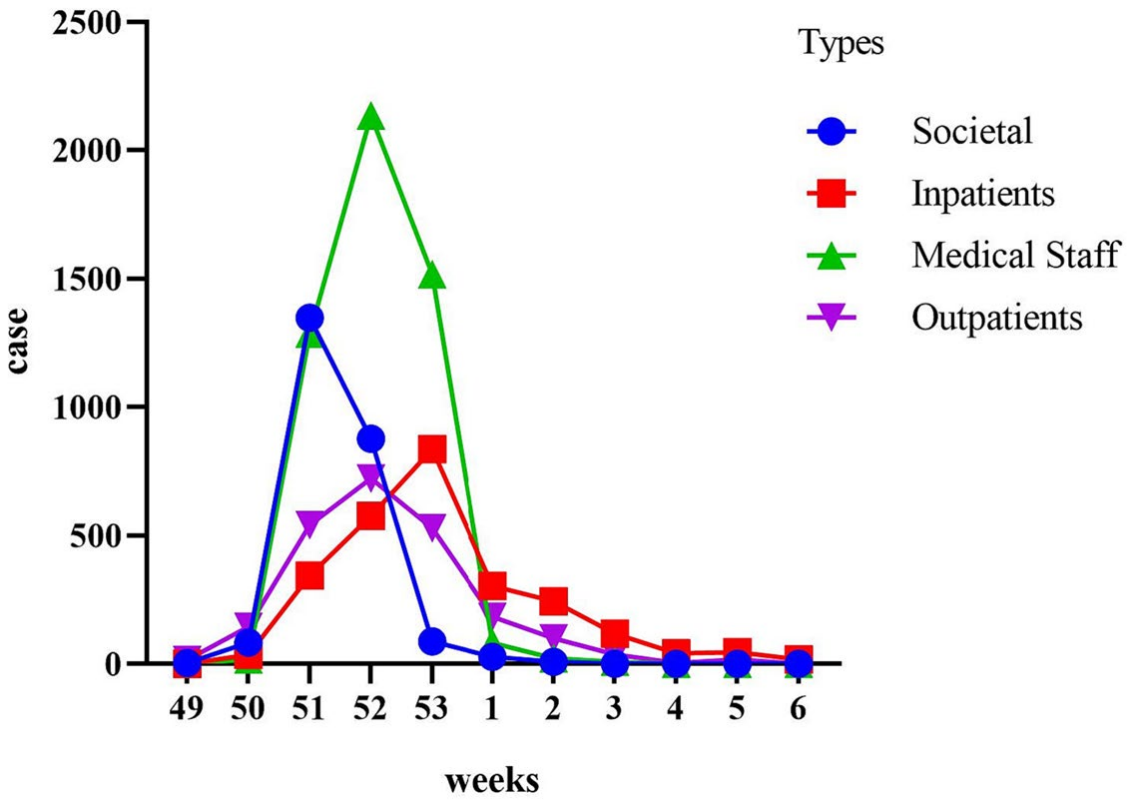
Time course of cumulative incidence in different populations. The populations are categorized as the Societal group, the Medical Staff group, the Outpatients group, and the Inpatients group. The study aims to observe the trend of positive cases within each population every week.

Our temporal analysis demonstrated that the initial impact of the Omicron outbreak was on the community population, followed by healthcare workers, outpatient attendees, and hospitalized patients (Figure 3). Within the community population, the incidence rate peaked in the third week of December, with 1970 cases per ten thousand individuals. Healthcare workers reached their peak in the fourth week of December, with 4,100 cases per ten thousand individuals. The third peak occurred in outpatient attendees, during the fourth week of December, with 2070 cases per ten thousand individuals. The final peak was observed in hospitalized patients during the last week of December, with 1,850 cases per ten thousand individuals.

### Distribution of Omicron positive cases by gender and age, and viral load analysis in different age groups

A total of 55,296 patients underwent SARS-CoV-2 NAT from December 1, 2022, to January 31, 2023, with 12,346 cases confirmed as positive. Among the positive cases, 27 were diagnosed with severe pneumonia and subsequently identified through metagenomic next-generation sequencing (mNGS) as Omicron. According to data from the China National GeneBank,^1^ Omicron was considered the predominant variant associated with the virus, accounting for over 95% of the cases. Table 1 presents the key epidemiological data of SARS-CoV-2-positive patients. The included patients numbered 55,296, with 25,097 males and 30,199 females. Among males, 5,366 tested positive (21.4%), while among females, 6,980 tested positive (23.1%). A chi-square test was conducted to validate gender differences in the overall dataset, yielding a value of *p* < 0.0001. The average age of positive cases was 43.4 years. The 20–29 age group had the highest number of cases (24.6%), followed by the 30–39 age group (22.5%), while the 0–9 age group had the fewest number of cases (1.42%). Ct values were unavailable for 13.4% of cases in the initial positive tests, but subsequent tests provided Ct values for both the N gene and ORF gene targets. Among the 12,346 positive cases, 10,730 (86.91%) had Ct values for the N gene, and 10,716 (86.80%) had Ct values for the ORF gene, so 86.91% of the positive cases had Ct values. Analysis using symmetric measurements demonstrated a high concordance of approximately 96.86% between the N gene and ORF gene, indicating a strong agreement (*p* < 0.0001). A chi-square test revealed a significant difference between the N gene and ORF gene (*p* < 0.0001). Pairwise Wilcoxon signed-rank tests indicated that Ct values for the N gene were significantly lower than those for the ORF gene, suggesting higher sensitivity of the N gene compared to the ORF gene.

**Table 1:** Basic epidemiologic data of SARS-CoV-2 positive individuals.

	*n* (%)	*x* ^2^	*p* value
*Total number*	12,346		
Females	6,980 (56.5%)	25.12	<0.001
Males	5,366 (43.5%)		
Mean age (median; IQR)	43.35 (39)		
*Age group*			
0–9	176 (1.4%)		
10–19	249 (2.0%)		
20–29	3,036 (24.6%)		
30–39	2,770 (22.4%)		
40–49	1822 (14.8%)		
50–59	1829 (14.8%)		
60–69	1,107 (9.0%)		
70–79	748 (6.1%)		
80+	609 (4.9%)		
*N gene Ct value*			
≤20	182 (1.5%)		
>20 ≤ 30	3,981 (32.2%)		
>30	6,529 (52.9%)		
No records	1,653 (13.4%)		
*ORF gene Ct value*			
≤20	116 (0.9%)		
>20 ≤ 30	3,456 (28.0%)		
>30	7,120 (57.7%)		
No records	1,654 (13.4%)		

*X*^2^: Pearson‘s Chi-squared test between male and female, *p* < 0.001. During periods of high incidence, contact tracking information is particularly challenging, resulting in incomplete records.

**Figure 4:**
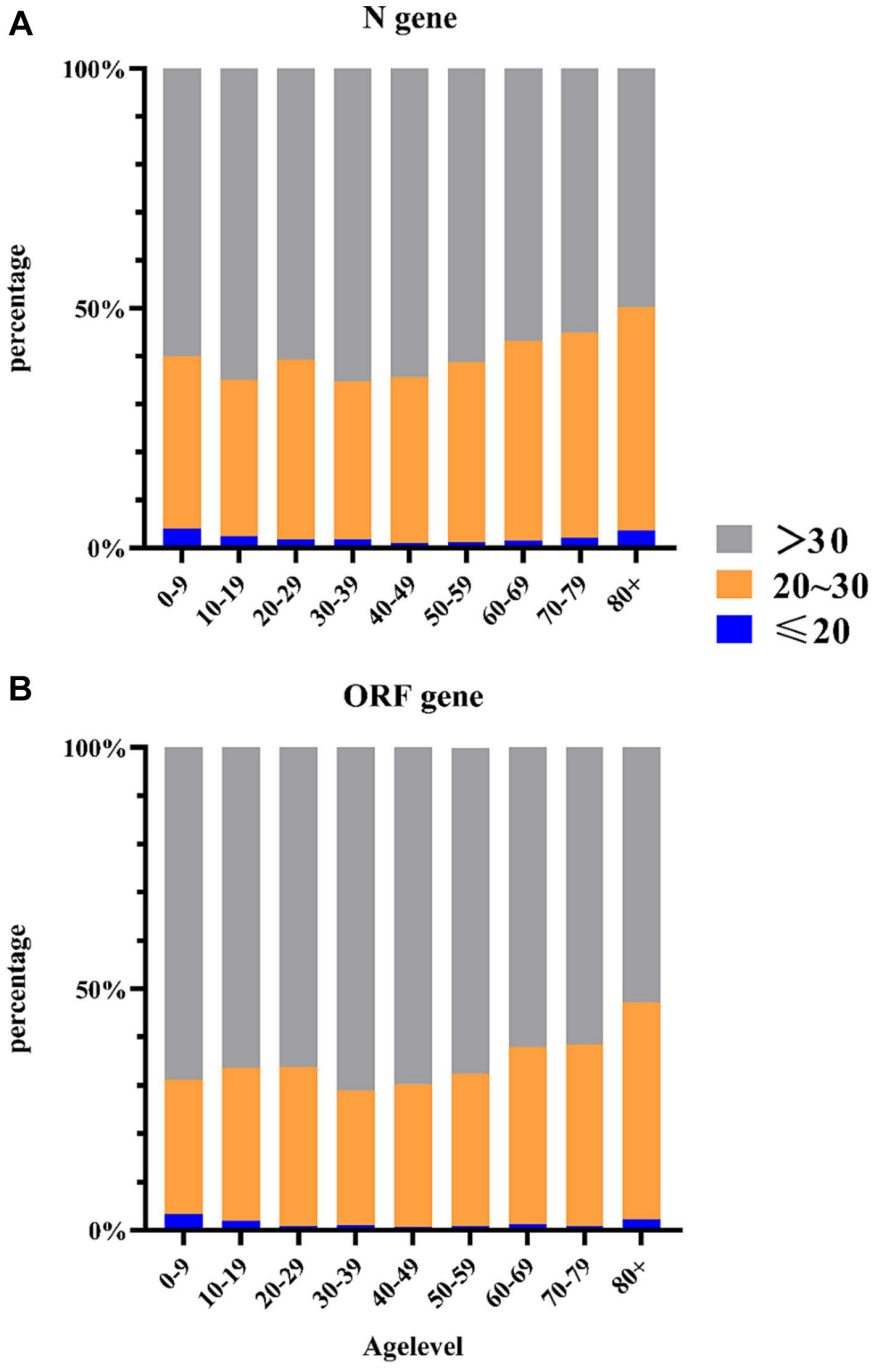
Distribution of Ct value categories (%) per age group. **(A)** Distribution of N gene Ct value categories (%) per age group. **(B)** Distribution of ORF gene Ct value categories (%) per age group.

Therefore, further investigations will focus on the Ct values of the N gene. We stratified the categories of N gene Ct values by age groups (Figure 4) and found differences in Ct values between age groups using Pearson’s chi-squared test analysis (*X*^2^ = 98.83, *p* < 0.0001). The proportion of cases with Ct values ≤20 was 4.05% for individuals aged 0–9 years, while >38.51% had values between 20 and 30. For individuals aged 30–39 years, 67.48% had Ct values >30. Older adults aged ≥80 years and children aged 0–9 years had a higher proportion of viral load (Ct value ≤20: 4.05% vs. 3.33%; Ct value >20 and ≤30: 38.51% vs. 45.18%). Similar results were found for ORF genes using Pearson’s chi-squared test analysis (*X*^2^ = 105.18, *p* < 0.0001). The proportion of cases with Ct values ≤20 was 4.05% for individuals aged 0–9 years, while >29.05% had values between 20 and 30. For individuals aged 30–39 years, 71.56% had Ct values >30. Older adults aged ≥80 years and children aged 0–9 years had a higher proportion of viral load (Ct value ≤20: 4.05% vs. 3.33%; Ct value >20 and ≤ 30: 38.51% vs. 41.33%). Due to the influence of age on Ct values, we will further analyze the differences in Ct values among different age groups.

### Differences in Ct values between age groups and viral load in Omicron-positive patients

An analysis of variance (ANOVA) was conducted to assess differences in Ct values between N genes (Table 2) or ORF genes (Table 3) and each age group. The results showed significant differences in Ct values among all age groups (*p* = 0.001 or *p* < 0.0001). When the age group >80 years was used as the control group, Ct values were significantly different from those of the group aged 20–59 years, with all Ct values significantly higher. This suggests that the number of viral loads was low after infection in the group aged 20–59 years, possibly due to their strong immune mechanisms. In contrast, older adults and children had a higher viral load, indicating a need for greater attention to infections in these age groups.

**Table 2:** Category distribution of N gene Ct values for each age group.

	Target 1 (N gene)							
	*n*	Mean	Median	SD	SE	IQR	*F*	*p* value
*Overall*	10,730	31.28	8.13	0.08	31.70	28–34.9	3.15	0.001
*Sex*								
Male	4,672	31.18	31.65	6.37	0.09	28–34.8	−1.15	0.249
Female	6,058	31.36	31.80	9.26	0.12	28.1–35		
*Groups*								
<10	148	30.61	31.01	0.44	31.55	27.65–34.375	−0.89	1.000
10 ~ 19	203	31.14	31.50	0.34	31.60	28.3–34.7	−1.81	1.000
20 ~ 29	2,571	31.25	31.40	0.15	31.60	28–34.8	−3.50	0.017
30 ~ 39	2,411	31.71	32.00	0.18	32.10	28.9–35.1	−4.68	<0.0001
40 ~ 49	1,586	31.43	31.79	0.11	32.00	28.575–35	−3.76	0.006
50 ~ 59	1,591	31.30	31.35	0.22	31.60	28–34.7	−3.44	0.021
60 ~ 69	966	31.13	31.19	0.35	31.20	27.675–34.7	−2.79	0.189
70 ~ 79	683	30.98	30.80	0.45	31.10	26.8–34.8	−2.27	0.847
>80	571	29.94	30.25	0.22	30.25	26.5–34		

**Table 3:** Category distribution of ORF gene Ct values for each age group.

	Target 2 (ORF gene)							
	*n*	Mean	Median	SD	SE	IQR	*F*	*p* value
*Overall*	10,716	31.97	4.68	0.05	32.50	29–35.8	8.93	<0.0001
*Sex*								
Male	4,668	31.95	32.40	4.64	0.07	28.9–35.6	−0.36	0.722
Female	6,048	31.99	32.50	4.71	0.06	29–35.9		
*Groups*								
<10	148	31.50	31.98	0.44	32.25	28.65–35.3	−1.46	0.872
10 ~ 19	203	31.91	32.30	0.33	32.80	29.1–35.5	−2.84	0.107
20 ~ 29	2,568	31.95	32.30	0.09	32.40	29–35.6	−4.93	<0.0001
30 ~ 39	2,409	32.42	32.83	0.09	33.00	29.6–36.05	−6.84	<0.0001
40 ~ 49	1,582	32.28	32.65	0.11	32.90	29.3–36	−6.06	<0.0001
50 ~ 59	1,588	31.89	32.20	0.11	32.30	29–35.4	−4.48	<0.0001
60 ~ 69	964	31.66	32.07	0.16	32.00	28.425–35.7	−3.24	0.033
70 ~ 79	683	31.47	31.65	0.19	32.10	27.7–35.6	−2.34	0.320
>80	571	30.79	31.05	0.22	31.00	27.1–35		

SD, standard deviation; SE, standard error; IQR, interquartile range. The gender group used a *T*-test with homogeneous variance; Analysis of variance was used for age groups. Target 1 had homogeneous variances, paired comparisons used the Bonferroni test, Target 2 had uneven variances, and paired comparisons used the Brown-Forsythe test, and pairwise comparisons used the Games-Howell test.

### Analysis of the correlation between Omicron viral load and laboratory biomarkers in hospitalized patients

To further explore the correlation between the number of viral loads in the body and laboratory biomarkers, we collected laboratory biomarker samples from patients who were hospitalized. These samples gave us a detailed set of laboratory indicators.

In this retrospective study, we analyzed laboratory indicators and blood cell counts of 2,363 hospitalized patients infected with Omicron using the Wilcoxon rank sum test to assess the differences between the N gene Ct value and these parameters (Table 4). Our results indicate that there were statistically significant differences (*p* < 0.05) in neutrophil percentage (NEUT%), eosinophil absolute (EO#), basophil absolute (BASO#), basophil percentage (BASO%), lymphocyte absolute (LY#), C-reactive protein (CRP), prothrombin time (PT), international standard ratio (INR), fibrinogen (FIB), aspartate aminotransferase (AST), lactate dehydrogenase (LDH), and B-type natriuretic peptide (BNP) between level 2 and level 3. In addition, the chi-square test showed that there was no statistically significant difference in gender between the Ct value level 2 and level 3 groups. In addition, our analysis suggests that as the viral load decreases, eosinophil percentage (EO%) decreases (from 0.12 to 0.05%), while inflammatory markers increase: CRP (from 11.51 mg/L to 32.97 mg/L), LDH (from 42 U/L to 257.00 U/L), BNP (from 61.50 pg./mL to 510.00 pg./mL), and fibrinogen (FIB) also increase (from 3.73 g/L to 4.25 g/L). These results are different from previously published findings, therefore, we further investigated whether there is a weak correlation between these laboratory indicators and Ct values.

**Table 4:** Wilcoxon rank-sum test between each N gene level group.

N level median (P25–P75)						
Variable	*n* (%)	Normal range	Level 1 (*n* = 39)	Level 2 (*n* = 872)	Level 3 (*n* = 1,328)	*p* value
WBC (x10^9^/L)	2,183 (97.49%)	4.00–10.00	5.970 (4.600–8.540)	6.800 (4.875–9.340)	6.700 (4.930–9.345)	0.33^a^
						0.326^b^
						0.904^c^
NEUT# (x10^9^/L)	2,183 (97.49%)	1.80–5.40	3.790 (2.770–6.330)	4.510 (2.995–7.395)	4.690 (3.075–7.390)	0.375^a^
						0.316^b^
						0.499^c^
NEUT%	2,183 (97.49%)	0.460–0.750	0.681 (0.613–0.824)	0.720 (0.600–0.833)	0.741 (0.618–0.844)	0.564^a^
						0.232^b^
						0.030^c^ *
EO# (x10^9^/L)	2,183 (97.49%)	0.05–0.50	0.060 (0.010–0.190)	0.060 (0.010–0.150)	0.030 (0.010–0.110)	0.824^a^
						0.233^b^
						0.0001^c^**
EO%	2,183 (97.49%)	0.005–0.050	0.012 (0.001–0.037)	0.010 (0.001–0.023)	0.005 (0.001–0.017)	0.587^a^
						0.105b
						0.0001^c^ **
BASO# (x10^9^/L)	2,183 (97.49%)	0.00–0.10	0.020 (0.000–0.040)	0.010 (0.010–0.030)	0.010 (0.000–0.020)	0.367^a^
						0.097^b^
						0.0001^c^ **
BASO%	2,183 (97.49%)	0.000–0.010	0.003 (0.001–0.005)	0.002 (0.001–0.004)	0.002 (0.001–0.004)	0.166^a^
						0.040^b^ *
						0.003^c^ **
MO# (x10^9^/L)	2,183 (97.49%)	0.00–0.50	0.470 (0.390–0.530)	0.500 (0.345–0.690)	0.480 (0.330–0.660)	0.643^a^
						0.995^b^
						0.111^c^
MO%	2,183 (97.49%)	0.030–0.080	0.078 (0.058–0.093)	0.075 (0.054–0.098)	0.072 (0.051–0.096)	0.782^a^
						0.444^b^
						0.085^c^
LY# (x10^9^/L)	2,183 (97.49%)	1.00–3.30	0.940 (0.700–1.730)	1.100 (0.615–1.635)	0.980 (0.565–1.545)	0.838^a^
						0.464^b^
						0.034^c^ *
LY%	2,183 (97.49%)	0.190–0.470	0.222 (0.095–0.269)	0.172 (0.086–0.278)	0.155 (0.086–0.262)	0.63^a^
						0.295^b^
						0.065^c^
PLT (x10^9^/L)	2,183 (97.49%)	100–300	205 (145.000–276.000)	207 (151.000–272.500)	205.000 (147.000–274.500)	0.911^a^
						0.814^b^
						0.685^c^
PCT (ng/L)	2,180 (97.49%)	0.00–0.05	0.172 (0.129–0.230)	0.187 (0.140–0.238)	0.183 (0.134–0.240)	0.653^a^
						0.74^b^
						0.624^c^
CRP (mg/L)	1924 (85.93%)	0.00–10.00	11.510 (5.560–63.795)	21.885 (5.152–77.980)	32.970 (6.610–97.505)	0.654^a^
						0.218^b^
						0.004^c^ **
APTT (s)	2046 (91.38%)	28.0–43.0	30.700 (27.450–36.550)	31.700 (28.300–37.725)	32.600 (28.600–38.100)	0.451^a^
						0.241^b^
						0.111^c^
PT (s)	2046 (91.38%)	11.0–14.0	12.050 (11.125–13.700)	12.700 (11.600–14.100)	13.000 (11.700–14.300)	0.165^a^
						0.062^b^
						0.024^c^ *
INR	2046 (91.38%)	0.80–1.15	0.980 (0.912–1.103)	1.020 (0.950–1.110)	1.030 (0.960–1.130)	0.118^a^
						0.042^b^ *
						0.030^c^ *
TT (s)	2046 (91.38%)	14.0–21.0	17.650 (17.100–18.275)	17.900 (17.100–18.800)	18.000 (17.200–18.900)	0.245^a^
						0.156^b^
						0.34^c^
FIB (g/L)	2046 (91.38%)	2.00–4.00	3.725 (2.882–4.938)	3.915 (2.900–4.920)	4.250 (3.178–5.300)	0.767^a^
						0.164^b^
						0.0001^c^ **
D.Dimer (mg/L FEU)	1,622 (72.44%)	0.00–0.55	1.235 (0.677–2.527)	1.130 (0.500–2.453)	1.180 (0.520–2.615)	0.655^a^
						0.891^b^
						0.326^c^
AST (U/L)	2,153 (96.15%)	1–37	27.000 (19.000–43.000)	28.000 (21.000–44.000)	31.000 (22.000–47.000)	0.642^a^
						0.291^b^
						0.012^c^ *
ALT (U/L)	2,153 (96.15%)	1–40	21.000 (12.000–29.000)	20.000 (14.000–34.000)	21.000 (15.000–34.000)	0.78^a^
						0.571^b^
						0.146^c^
LDH (U/L)	1829 (81.68%)	114–240	242.000 (171.000–317.000)	243.000 (193.000–328.000)	257.000 (198.000–351.250)	0.641^a^
						0.302^b^
						0.020^c^ *
IL-6 (pg/mL)	665 (29.70%)	0.00–0.07	33.150 (15.670–47.130)	26.845 (8.018–76.543)	25.765 (7.160–88.625)	0.814^a^
						0.841^b^
						0.966^c^
CK (U/L)	532 (23.76%)	0.10–4.94	78.000 (52.000–300.500)	90.000 (50.750–246.250)	84.000 (42.000–183.000)	0.946^a^
						0.754^b^
						0.142^c^
CKMB (ng/L)	1,486 (66.36%)	0.10–4.94	1.680 (0.950–2.690)	1.830 (0.980–3.158)	1.650 (0.890–3.120)	0.705^a^
						0.912^b^
						0.344^c^
TNT (ng/L)	1,486 (66.36%)	0.000–0.014	0.023 (0.009–0.042)	0.018 (0.009–0.044)	0.022 (0.010–0.046)	0.612^a^
						0.91^b^
						0.11^c^
MYO (ng/L)	1,486 (66.36%)	25.00–75.00	65.000 (30.700–162.500)	58.050 (25.825–177.500)	57.300 (28.400–163.000)	0.727^a^
						0.841^b^
						0.678^c^
BNP (pg/mL)	1,503 (67.12%)	0.00–10.00	561.500 (79.125–1943.750)	379.000 (93.125–1616.750)	510.000 (132.000–2159.000)	0.974^a^
						0.501^b^
						0.010^c^ **

*p < 0.05, **p < 0.01; ^a^Compare N gene level 1 and N gene level 2 with the non-parametric test. ^b^Compare N gene level 1 and N gene level 3 with the non-parametric test. ^c^Compare N gene level 2 and N gene level 3 with the non-parametric test.

WBC, white blood cell; NEUT#, Absolute number of Neutrophils; NEUT%, Percentage of Neutrophils; EO#, Absolute number of Eosinophils; EO%, Percentage of Eosinophils; BASO#, Absolute number of Basophils; BASO%, Percentage of Basophils; MO#, Absolute number of Monocytes; MO%, Percentage of Monocytes; LY#, Absolute number of Lymphocytes; LY%, Percentage of Lymphocytes; PLT, Platelet; CRP, C-reactive protein; APTT, Activated partial thromboplastin time; PT, Prothrombin time; TT, Thrombin time; INR, International standardized ratio of Prothrombin time; FIB, Fibrinogen; AST, Aspartate Transaminase; ALT, Alanine aminotransferase; LDH, Lactate dehydrogenase; IL-6, Interleukin-6; CK, Creatine kinase; CK-MB, Creatine kinase isoenzyme-MB; TNT, Troponin T; MYO, Myoglobin; BNP, N-terminal pro-B-type natriuretic peptide.

Spearman correlation analysis showed weak correlations between Ct value and laboratory parameters (Table 5), such as CRP (*r_s_* = 0.059, *p* = 0.009), PT (*r_s_* = 0.057, *p* = 0.009), INR (*r_s_* = 0.055, *p* = 0.013), TT (*r_s_* = 0.051, *p* = 0.020), FIB (*r_s_* = 0.087, *p* < 0.001), AST (*r_s_* = 0.067, *p* = 0.002), ALT (*r_s_* = 0.045, *p* = 0.036), LDH (*r_s_* = 0.078, *p* = 0.001), and BNP (*r_s_* = 0.063, *p* = 0.014).

**Table 5:** Spearman analysis of the correlation between the Ct value of the N gene and serological indicators.

Variable	*n* (%)	Correlation coefficient (r)	*p* value
WBC (x10^9^/L)	2,183 (97.49%)	0.018	0.405
NEUT# (x10^9^/L)	2,183 (97.49%)	0.039	0.068
NEUT%	2,183 (97.49%)	0.082**	<0.001
EO# (x10^9^/L)	2,183 (97.49%)	−0.118**	<0.001
EO%	2,183 (97.49%)	−0.119**	<0.001
BASO# (x10^9^/L)	2,183 (97.49%)	−0.093**	<0.001
BASO%	2,183 (97.49%)	−0.083**	<0.001
MO# (x10^9^/L)	2,183 (97.49%)	−0.042*	0.048
MO%	2,183 (97.49%)	−0.058**	0.006
LY# (x10^9/^L)	2,183 (97.49%)	−0.065**	0.003
LY%	2,183 (97.49%)	−0.069**	0.001
PLT (x10^9^/L)	2,183 (97.49%)	0.004	0.841
PCT (ng/L)	2,180 (97.49%)	0.005	0.823
CRP (mg/L)	1,924 (85.93%)	0.059**	0.009
APTT (s)	2,046 (91.38%)	0.022	0.331
PT (s)	2,046 (91.38%)	0.057**	0.009
INR	2,046 (91.38%)	0.055*	0.013
TT (s)	2,046 (91.38%)	0.051*	0.020
FIB (g/L)	2,046 (91.38%)	0.087**	<0.001
D.Dimer(mg/L FEU)	1,622 (72.44%)	0.022	0.367
AST (U/L)	2,153 (96.15%)	0.067**	0.002
ALT (U/L)	2,153 (96.15%)	0.045*	0.036
LDH (U/L)	1,829 (81.68%)	0.078**	0.001
IL-6 (pg/mL)	665 (29.70%)	−0.023	0.560
CK (U/L)	532 (23.76%)	−0.063	0.152
CKMB (ng/L)	1,486 (66.36%)	−0.012	0.645
TNT (ng/L)	1,486 (66.36%)	0.045	0.092
MYO (ng/L)	1,486 (66.36%)	−0.008	0.772
BNP (pg/mL)	1,503 (67.12%)	0.063*	0.014

*p < 0.05, **p < 0.01.

Spearman correlation analysis also showed that the Ct value was weakly correlated with NEUT% (*r_s_* = 0.082, *p* < 0.0001); with EO# (*r_s_* = −0.118, *p* < 0.0001), EO% (*r_s_* = −0.119, *p* < 0.0001), BASO# (*r_s_* = −0.093, *p* < 0.0001), BASO% (*r_s_* = −0.083, *p* < 0.0001), MO# (*r_s_* = −0.042, *p* = 0.048), MO% (*r_s_* = −0.058, *p* = 0.006), LY# (*r_s_* = −0.065, *p* = 0.003), and LY% (*r_s_* = −0.069, *p* = 0.001).

## Discussion

On December 1, 2022, approximately 35 months after the first reported case of COVID-19, and following an estimated 640 million cases and 660,000 deaths worldwide (32), a new SARS-CoV-2 variant known as Omicron emerged, bringing with it a new wave of infections that have sometimes affected the entire world (33). Understanding the epidemiology of the virus and the factors associated with its transmission and severity is critical for effective control and prevention measures (12, 34). However, although there has been a rapid spread of the virus, a rapid decline in cases has also been observed, particularly in regions where Omicron is endemic, such as South Africa (35). Given the incomplete epidemiological data from large-scale Omicron pandemics in the South China region, our study would be helpful to fill this gap. Typically, significant changes in laboratory indicators are indicative of the severity of a patient’s illness. However, the relationship between Ct values of Omicron and laboratory indicators is not well established, which is also evaluated in this study. Our results also show the time course of cumulative incidence. In our analysis of the weekly cumulative infection positive rate and the weekly positive rate for each age group during the outbreak, we found that the prevalence of Omicron experienced a rapid peak and decline within 6 weeks. As Omicron has spread on a large scale for the first time in Guangzhou, and it has been reported that the spread is closely related to age (36), gender (37), protective measures (38), and other factors, we conducted this retrospective analysis on patients in our hospital.

In this study, we reported 12,346 (22.3%) positive cases of COVID-19 out of 55,296 samples. As a point of comparison, a study of 556 passengers on a flight from two Chinese provinces in Italy that took place in late December 2022 reported that 126 (22.7%) of those passengers tested positive for SARS-CoV-2, similarly to our study (39). We have also shown that Omicron infection affects females more than males, with a ratio of 56.5% vs. 43.5%, respectively. This trend is consistent with the results of a previous study on the Omicron outbreak in Shanghai, which showed 54.7% for females and 45.3% for males (40). This may be due to the higher proportion of female medical staff, for example, women accounted for 56% of the medical staff assisting Hubei in Guangzhou, and they often play the role of caregivers and front-line healthcare workers in the family.

We investigated the epidemiology of Omicron infection in hospitalized patients. Our results showed that the highest number of positive cases was observed in the 20–39 age group, consistent with previous studies (41). Similarly, in Hohhot, there was a wide age range among COVID-19 patients, spanning from 3 days to 89 years. The 30–59 age group was the primary affected population in this outbreak, accounting for 53.74% of the total cases (42). This may be related to the active social activity of this age group, indicating the need to reduce social activities during an Omicron outbreak. In contrast, children aged 0–9 years had the fewest cases, likely due to parents’ protective measures and less contact with social activities. This finding is consistent with previous studies showing that children are less susceptible to SARS-CoV-2 (43, 44) and may also be related to the government’s effective and strict protection measures for children.

In addition to epidemiological data, nucleic acid detection targets N and ORF genes, and reporting the Ct value in test results is important (45). Generally, a low Ct value indicates a higher viral load within the patient, making them potentially more contagious and having higher infectivity. Patients with high Ct values may have a lower transmission risk. Some studies have shown a correlation between viral carrier amount and Ct value, with lower Ct values indicating higher viral loads (46, 47). The findings indicated that more than 50% of patients had a Ct value >30, suggesting a relatively low viral copy number and potentially mild symptoms. Our results revealed no significant difference in Ct values between genders, which is consistent with Jessica Penney’s report (48). However, there was a significant variation in Ct values among different age groups. The older adult (>60 years) and children (<19 years) exhibited lower Ct values, aligning with previous research on respiratory viruses. Overall, these results suggest that older adults and children infected with Omicron had higher viral loads and that there may be age-related differences in viral loads. They have been reported to generally have higher viral loads and longer viral shedding times, making them more infectious (49, 50). Hence, schools and senior care facilities should take extra precautions to prevent and monitor virus spread. The higher viral load in older adults and children may increase their likelihood of transmitting the virus, which highlights the importance of protecting and managing this group after Omicron infection to minimize infectivity to others.

Due to the diversity of population sources, we conducted an epidemiological analysis of the disease from three dimensions: age, gender, and occupational groups. Regarding age, we examined infection rates and peak times among different age groups. We found that individuals aged 30–49 had the highest infection rate, likely due to their extensive social activities, while children aged 0–9 had the lowest infection rate, possibly due to protective measures for children and government interventions. In terms of gender, we analyzed infection rates among different genders and observed that females had a higher infection rate. This could be attributed to the predominance of female healthcare professionals or potentially lower immune responses in females. Finally, considering occupational differences, we divided the patients into four groups: Societal, Medical Staff, Outpatients, and Inpatients groups. Analysis of the results showed that the peak time for Omicron infection differed among the groups. The Society group experienced the earliest peak, likely due to their general susceptibility to the virus. In contrast, with their professional habits and knowledge, the Medical Staff group was better equipped to take effective protective measures, resulting in a later peak time compared to the Society group. Outpatients experienced a peak time later than that of the Society group, as patients often manage their infection before seeking medical treatment and only visit hospitals when their symptoms become severe. The peak time was delayed for hospitalized patients who tested positive for Omicron due to the strict infection control measures implemented in the hospital. Our findings highlight the significance of increasing COVID-19 awareness among the general population and improving their medical literacy and prevention knowledge to help curb the transmission of the virus (51, 52). Although virus contact and infection are inevitable, patients in the ward receive the best protection, as demonstrated by the delayed peak time of infection compared to other groups, even in the context of a major outbreak.

According to reports (53), the Da An’s test kit has been found to exhibit higher sensitivity toward the N gene compared to the ORF1ab gene, both at low and medium concentrations of the reference sample. Additionally, as Sylvain Robinet and his colleagues found in COVID-19 NAT, the Ct values of the novel coronavirus N gene are typically lower than those of the ORF gene. This study, based on follow-up data from over 67,000 patients, suggests that the N gene may serve as a sensitive indicator for detecting new active viral circulation (54). Interestingly, our results also indicate that N genes have higher sensitivity than ORF genes, so using the N gene for detection is more helpful for early diagnosis. Therefore, we will proceed with further research utilizing the Ct values of the N gene.

In this study, we aimed to investigate the relationship between Ct values and laboratory indicators in hospitalized patients. The availability of comprehensive laboratory indicators allowed us to explore this association and analyze the statistical differences, which have not been previously reported. Notably, we found statistically significant differences in complete blood counts (NEUT, EO, BASO, LY) (55, 56), CRP, PT (57, 58), FIB, AST, LDH, and BNP between Ct values of 20–30 and > 30. These findings suggest that the Ct value may serve as an important indicator for assessing the immune system response and health condition of patients. To analyze the relationship between laboratory indicators and the N gene Ct value, we employed the Wilcoxon rank-sum test. In addition to the previous information, we conducted further investigations into the correlation between Ct values and laboratory indexes. Previous studies have highlighted the significance of disease severity and clinical biomarkers in understanding the severity of COVID-19. Our findings revealed a weak correlation (*r*_s_ < 0.2) between the Ct values of the N gene and the laboratory’s related indicators. Typically, lower Ct values are associated with more pronounced abnormalities in laboratory indexes, indicating a higher disease severity (59, 60). However, our extensive data analysis suggests that the severity of the disease cannot be directly inferred from Ct values. These results align with the research conducted by Padoan A. demonstrated no difference in the average viral load between patients with and without pneumonia (61). Our study supplements existing research by highlighting a relatively weak correlation between viral load and clinical laboratory testing results, suggesting that there may not be a strong association between viral load and abnormalities in the testing results. This phenomenon may be attributed to variations in individual immune system responses and the degree of tissue damage caused by Omicron infection. Some infected individuals exhibit significant abnormalities in laboratory indicators, while others show relatively normal laboratory results, despite similar viral copy numbers. These research findings emphasize the need for caution when interpreting laboratory test results for Omicron infection and highlight that the assessment of infection severity and intensity should not solely rely on viral copy numbers. Instead, a comprehensive evaluation of individual immune responses, inflammation regulation, and tissue damage should be considered to provide a more holistic understanding of the infection characteristics.

In conclusion, epidemiological data and the reporting of Ct values in test reports are both essential for effective public health responses. By providing accurate and timely data, we can identify patterns and trends in disease occurrence, monitor the spread of infectious diseases, and inform public health policy and interventions.

## Limitation

The retrospective study on the epidemiological and laboratory characteristics of Omicron infection in our hospital has several limitations that should be taken into consideration when interpreting the findings. Firstly, although the sample size was adequate for a single-center study, it is unclear if the findings can be generalized to other settings with different patient populations and demographics. Secondly, the Ct value was not recorded at the beginning of the study, which may have impacted the accuracy of the results. Additionally, for the sake of sampling convenience, throat swabs were employed for nucleic acid sampling, which is less precise than nasopharyngeal swabs. Lastly, the study included patients who met the criteria for COVID-19 infection, regardless of possible concurrent infections with other pathogens. Therefore, it is necessary to interpret the findings with caution, considering these limitations.

## Conclusion

Overall, these findings highlight the importance of ongoing monitoring and research into the Omicron variant. By understanding its behavior and characteristics, we can develop effective public health strategies to mitigate its impact on global health.

## Data availability statement

The original contributions presented in the study are included in the article/Supplementary material, further inquiries can be directed to the corresponding authors.

## Ethics statement

The studies involving humans were approved by IEC for Clinical Research and Animal Trials of the First Affiliated Hospital of Sun Yat-sen University. The studies were conducted in accordance with the local legislation and institutional requirements. The human samples used in this study were acquired from primarily isolated as part of your previous study for which ethical approval was obtained. Written informed consent for participation was not required from the participants or the participants’ legal guardians/next of kin in accordance with the national legislation and institutional requirements.

## Author contributions

JC: Writing – original draft, Data curation, Formal analysis, Methodology. YW: Formal analysis, Writing – original draft. HY: Formal analysis, Writing – original draft. RW: Formal analysis, Writing – original draft. XY: Data curation, Writing – original draft. HH: Data curation, Writing – original draft. LA: Data curation, Writing – original draft. TZ: Writing – original draft, Data curation. BH: Writing – original draft, Project administration. ML: Project administration, Writing – original draft. TD: Supervision, Writing – review & editing. YL: Supervision, Writing – review & editing. PC: Writing – review & editing, Project administration, Supervision.

## Footnotes

^1^ https://ngdc.cncb.ac.cn/ncov/
