# Supplementary material for: Epidemiological and laboratory characteristics of Omicron infection in a general hospital in Guangzhou: a retrospective study
Source: Front Public Health. 2023 Nov 29;11:1289668. doi: 10.3389/fpubh.2023.1289668 (PMC10716230; doi:10.3389/fpubh.2023.1289668)
Supplement: Supplementary file 1 [file Image_1.pdf]

Supplementary Figure

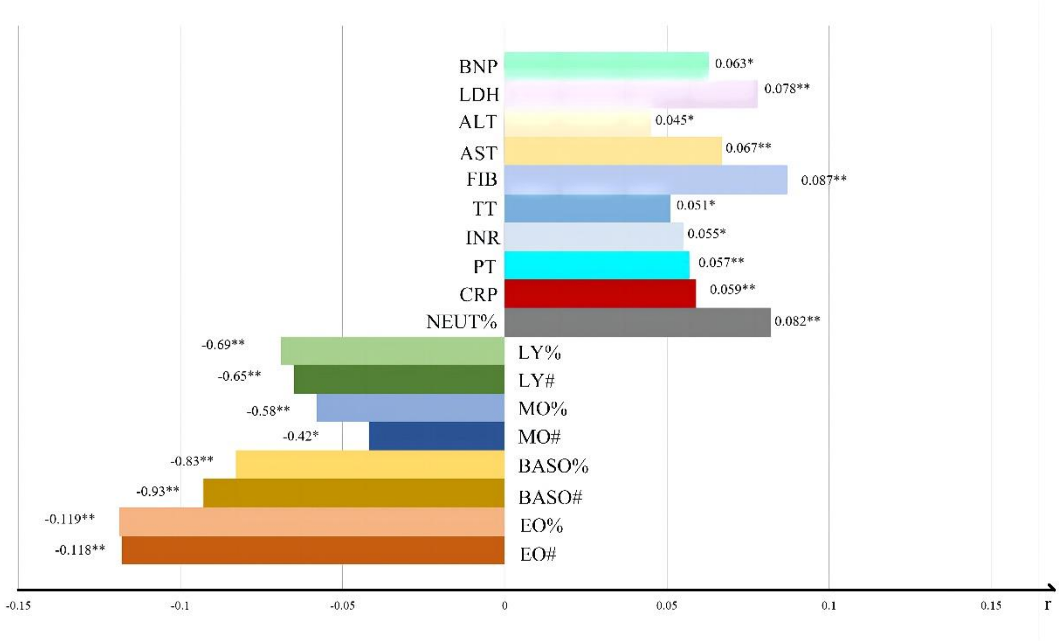

Supplementary Figure 1: Spearman correlation analysis coefficients and P values between Ct values of N genes and serological indices. * p <0.05, ** p <0.01
